# The dominant seagrass herbivore *Sarpa salpa* shifts its shoaling and feeding strategies as they grow

**DOI:** 10.1038/s41598-020-67498-1

**Published:** 2020-06-30

**Authors:** Xavier Buñuel, Teresa Alcoverro, Jordi F. Pagès, Javier Romero, Juan M. Ruiz, Rohan Arthur

**Affiliations:** 1 Centre d’Estudis Avançats de Blanes (CEAB-CSIC), Accés a la cala Sant Francesc 14, 17300 Blanes, Spain; 2 Departament de Biologia Evolutiva, Ecologia i Ciencies Ambientals, Facultat de Biologia, Universitat de Barcelona, Av. Diagonal, 643, 08028 Barcelona, Spain; 3 Nature Conservation Foundation, 3076/5, 4th Cross, Gokulam Park, Mysore, Karnataka 570 002 India; 4 Seagrass Ecology Group, Oceanographic Center of Murcia, Spanish Institute of Oceanography, C/Varadero, 30740 San Pedro del Pinatar, Murcia Spain

**Keywords:** Behavioural ecology, Ecological networks, Macroecology, Ecology

## Abstract

The relative benefits of group foraging change as animals grow. Metabolic requirements, competitive abilities and predation risk are often allometric and influenced by group size. How individuals optimise costs and benefits as they grow can strongly influence consumption patterns. The shoaling fish *Sarpa salpa* is the principal herbivore of temperate *Posidonia oceanica* seagrass meadows. We used in-situ observations to describe how ontogeny influenced *S. salpa* individual feeding behaviour, shoaling behaviour and group foraging strategies, and its potential consequences to seagrass meadows. Shoaling was strongly influenced by body length: shoals were highly length-assorted and there was a clear positive relationship between body length and shoal size. Foraging strategies changed dramatically with shoal size. Small shoals foraged simultaneously and scattered over large areas. In contrast, larger shoals (made of larger individuals) employed a potentially cooperative strategy where individuals fed rotationally and focused in smaller areas for longer times (spot feeding). Thus, as individuals grew, they increased their potential impact as well, not merely because they consumed more, but because they formed larger shoals capable of considerably concentrating their grazing within the landscape. Our results indicate that ontogenetic shifts in group foraging strategies can have large ecosystem-wide consequences when the species is an important ecosystem modifier.

## Introduction

While feeding in groups has clear immediate and evolutionary advantages, it is not without its challenges^1^. The benefits of locating resources, facilitating consumption and diluting predation risk have to be offset against strong competitive pressures within the group, particularly when resources are scarce^2,3^. For herbivores that spend a good portion of their time either trying to find food or feeding, group foraging has additional advantages. For one, it increases the success of finding feeding sites—either because there are more eyes to search, or because some individuals are better experienced^4^. Once a resource is found, cooperative feeding can also maximize the foraging efficiency of the group^5,6^. Feeding is a particularly risky activity in predator-prone areas, and groups help both to dilute the risk of individual predation as well to increase overall group vigilance^7–9^. However, as the size of the group increases, so do the costs of joining it^10^. For instance, the increased vigilance and dilution that groups provide needs to be balanced against higher conspicuousness as groups grow^11^; odd sized individuals or those in a weak physical state may be easy pickings in larger groups^12–14^. Also, in resource-limited environments, group foraging can enhance intraspecific competition^2,3^. How individuals optimise costs and benefits is linked to the size, composition and behaviour of the group^8,10^, and can determine how key functions are distributed across the habitat. Large herbivore aggregations can radically modify vegetation structure^15,16^, leading, in the extreme, to complete habitat collapse^15,17^. These dense aggregations lie at one end of a spectrum; many herbivores show remarkable flexibility in group size, from foraging alone to forming herds or schools of thousands of individuals^18^. Determining what characterises variability in group size and how it influences foraging decisions requires an understanding of how costs and benefits change with resource distribution, within-species interactions, and with an individual’s metabolic needs and abilities.

The costs and benefits of group living can alter through life since they scale strongly with individual size. Smaller individuals face considerably higher risks of predation^19,20^ and may respond by choosing either safety-in-numbers or inconspicuousness strategies by foraging in large or small groups respectively. As individuals grow, their susceptibility to predation typically reduces due to decreasing predator-prey body size relationships^21,22^. Many species may find size an ultimate refuge—with no predators serving a realistic threat beyond a threshold^23–26^. However, these larger individuals may still find it to their advantage to forage in groups if it increases their ability to find and access resources or if, despite having reached a size refuge, they still perceive predation a threat^27^. This may be particularly true when consumption scales with size or if there are major ontogenetic shifts in metabolic requirements as individuals age. Finally, smaller individuals may not be able to hold their own in competitive interactions with larger conspecifics in groups of mixed sizes and may find themselves at a disadvantage while foraging^12,13,28^, while larger individuals may face no such competitive pressure. Thus, body size can play an important role in the group-foraging decisions that individuals make.

**Figure 1:**
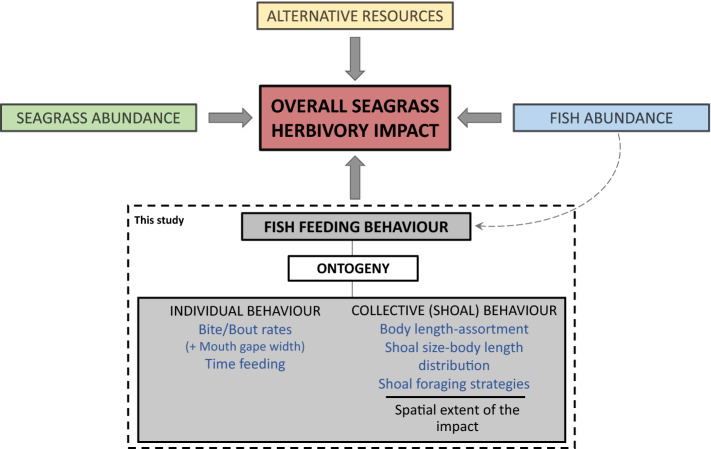
Factors influencing the total impact of herbivory on plant-dominated ecosystems like seagrass meadows. Our study focuses on unpacking how ontogeny influences individual and collective feeding behaviours.

The result of this dynamic individual decision-making is that group size can vary widely as a function of the composition of the population within an area^29^. This can have far-reaching implications for the landscape matrices within which herbivores forage^30,31^, particularly if the impact of foraging behaviour scales with group size. For instance, the size of a group may determine how it moves across the matrix, the time it spends within each patch and the time it spends foraging (as opposed to being vigilant, looking for other patches, etc.). In addition, increasing group size is often linked to cooperative feeding and higher degrees of specialisation, which might result in highly efficient forage extraction^32^. While evaluating the total impact of herbivory on a system, herbivore density, and the quality and quantity of resources (both primary and alternate) is essential (see Fig. 1). However, given how variable foraging decisions can be, the pattern and intensity of herbivory within a landscape may well be strongly influenced by group foraging strategies^15–17^.

We explored the effect of fish size in grouping and foraging behaviour in the fish *Sarpa salpa* in Mediterranean seagrass meadows. *S. salpa* is the primary vertebrate herbivore in shallow *Posidonia oceanica* seagrass meadows^33,34^ and rocky habitats^35,36^, often forming groups of hundreds of individuals^37^. It consumes a large proportion of annual primary production *of P. oceanica*^38^, which can drastically reduce seagrass canopies, impact plant fitness^39^, and result in cascading effects to other meadow-dwelling organisms, such as increasing predation risk^40^. In this paper, we used extensive field observations of *S. salpa* foraging shoals of different size classes in several meadows to explore how ontogeny influences individual feeding behaviour, shoal length-assortment, shoal size, shoaling feeding behaviour and shoal-specific impacts (Fig. 1).

## Methods

### Study system and study design

*Sarpa salpa* (Linnaeus 1758) is a demersal marine fish (Sparidae) that lives mostly in groups on sandy bottoms, rocky reefs and seagrass meadows from the surface to around 70 m^41^. It extends from the North Sea to the Cape of Good Hope, found in several locations from the Eastern Atlantic to the Western Indian Ocean (south of Mozambique), and is also abundant in the Black Sea and the Mediterranean^41,42^. It is one of few strictly herbivorous fish species in the Mediterranean Sea, basing its diet on macroalgae^36^ and the seagrass *Posidonia oceanica* (L.)^43^. It is a major consumer of *P. oceanica*, contributing to 75% of total herbivory consumption of the plant^38^. *S. salpa* food preferences change with age, with young individuals choosing a higher proportion of macroalgae, while adults tend to feed mainly on seagrass leaves^37,44^. Although adult *S. salpa* have few existing fish predators in the Mediterranean, juveniles and young fish may still experience predation pressure from large predatory fish. The species is not typically targeted by commercial fishing but does get extracted as bycatch by artisanal and recreational fishers^45–47^. As a result, its populations are often highest inside fishery reserves and Marine Protected Areas^47^.

**Figure 2:**
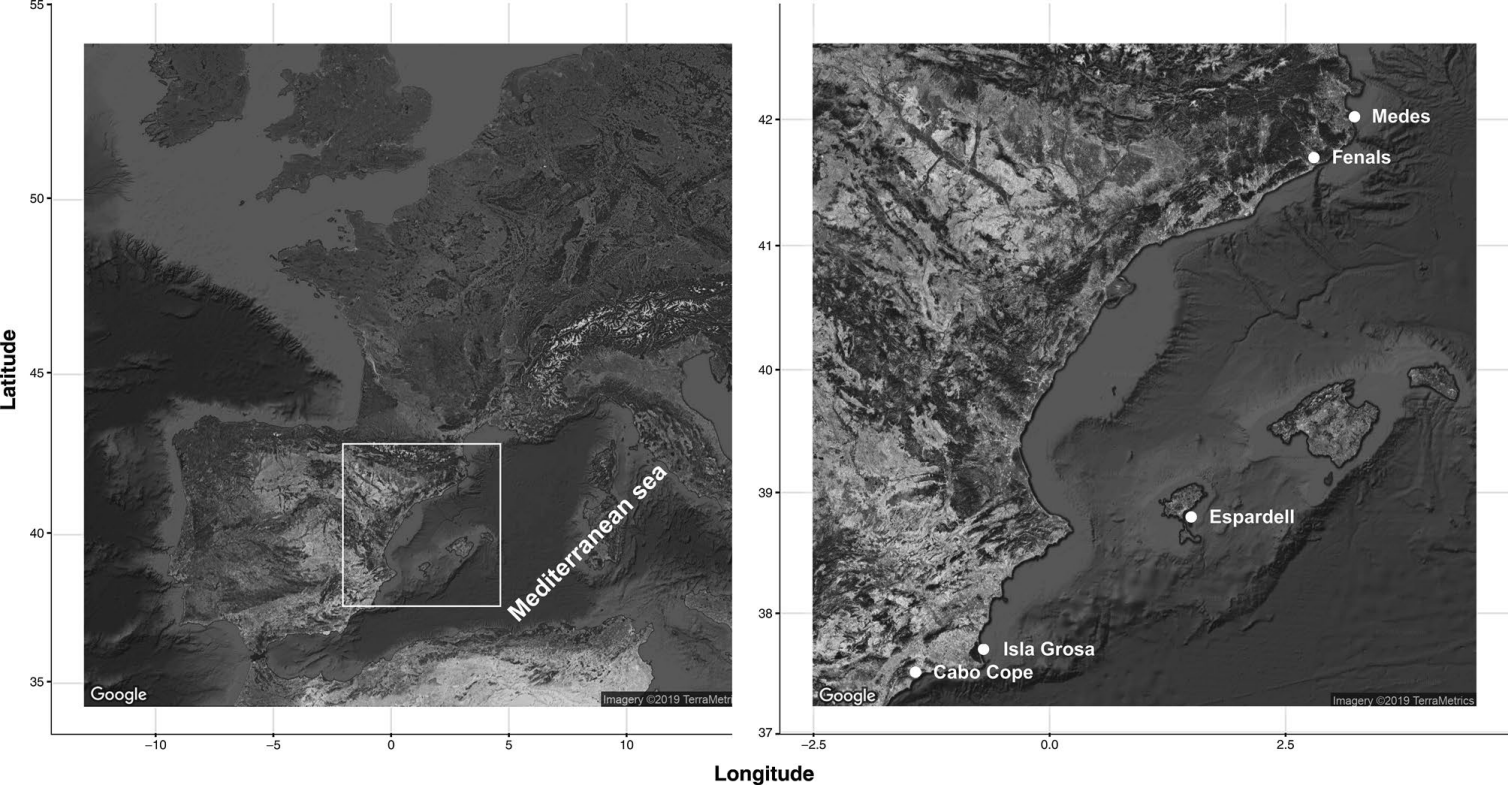
Distribution of study sites across the Spanish coast in the Western Mediterranean Sea. Sample sites are located across the Catalan coast, Balearic Islands and the coast of Murcia. Map was created using ggmap package in R software^72^ (R version 3.6.3 https://cran.r-project.org/bin/windows/base/).

To determine if *S. salpa* ontogeny influences its feeding and shoaling behaviour and describe its potential consequences on seagrass meadows, we conducted extensive field observations of *S. salpa* individuals that differed in their body lengths and studied their foraging behaviour (individual feeding activity, shoaling behaviour, group feeding strategies and potential impact, summarised in Fig. 1). We used fish length as a measure of ontogeny since there is a clear relationship between age and length in *S. salpa*^48^. The study was conducted in 5 locations dominated by seagrass meadows across the NW coast of Spain (Fig. 2), chosen for their high abundances of *S. salpa*^49^. Our observations were limited to shallow *P. oceanica* seagrass meadows (5–8 m). Shoals (or rare solitary individuals) were chosen as encountered in a random swim through the meadow (always maintaining the same depth and always within the meadow). Since all fish observed during our study shoaled in assorted sized groups (see "Results" section) we tracked the behaviour of entire shoals. We allowed a few minutes (3–4 min) for the shoal to acclimatise to the observer’s presence and then followed the shoal, recording it with a hand-held underwater video recorder (average of 7 min of footage per shoal). We stopped recording when the shoal travelled across large patches of sand or toward deeper areas. Across all locations we followed a total of 93 feeding shoals. Recordings differed in their level of observable detail and duration so some were excluded for analyses that required finer scale observation or were not long enough. However, all observations were considered to determine the overall *S. salpa* shoal distribution in each meadow (Fig. S1). Our observations were conducted in summer (June–August 2016), when feeding *S. salpa* are most active^38,50^. For each shoal we estimated group size (number of fish individuals per shoal) and used the fork length as a measure of individual body lengths (cm) within each shoal (from multiple randomly selected individuals). Shoal size was estimated by counting the total number of individuals in the shoal whenever the entire group was visible in a single frame. For large shoals (more than 50 individuals) several counts were conducted of different visible frames in order to reduce errors associated with estimating shoal size. Shoals were then classified based on the number of individuals (shoal size): small (< 15 individuals), medium (15–50 individuals), large (51–150 individuals) and very large (> 150 individuals). Within each shoal, the length of individuals was estimated from screen captures using the software ImageJ^51^. We used the width of *P. oceanica* leaves as a standard reference, given that they are relatively consistent in size (~ 1 cm); we only measured fish that were on the same plane as the leaf to avoid estimation error. Whenever possible, we attempted to measure 15–30% of individuals in each shoal assuming that it is an accurate representation of its composition. Shoals were also classified based on the average body length of the fish composing them: class 1 (< 14.6 cm), class 2 (14.6–20.2 cm), class 3 (20.3–26 cm) and class 4 (> 26 cm). In addition, for each observation (i.e. each video), we visually classified seagrass cover within three categories: continuous (100–80% of substrate covered by *P. oceanica*), fragmented (80–30%) and very fragmented (< 30%).

### Individual activity

We used three metrics to assess individual feeding: (1) bite rates per individual (number of bites per unit of time), (2) bout duration per individual (i.e. time each fish spent between its first descent into the seagrass leaves to feed and its exit towards the water column), and (3) number of bouts during the feeding time. Bite rate and bout assessments were done separately, since the quality of the video needed to accurately estimate bite rate was considerably higher than for determining if a fish was feeding within the meadow or not (bout duration and number of bouts). Therefore, for the bite rate analysis, we selected clips of any duration where the mouthparts of individual fish could be clearly seen. We were less restrictive with the quality of the video for estimates of bout duration and number, but sought a minimum video length (i.e. 30 s) in order to standardise our assessment. From 1 to 5 individuals were visually followed from each shoal to measure individual activity.

### Shoal composition

We analysed the shoal composition of *S. salpa,* to determine if they showed length-assorted grouping (i.e. individuals of the same length shoaling together). We used the average body length and coefficient of variation within each shoal as a measure of length-assorted grouping. In addition, we evaluated if shoal size (number of individuals) was influenced by average body length of shoal.

### Shoal foraging behaviour

We analysed shoal behaviour with the open-source event-logging software BORIS^52^, which is designed for video-coding behavioural observations and allows users to calculate the time allocated to different behavioural states. We defined three general behavioural states for the shoals assessed: ‘swimming’, ‘hovering’ and ‘feeding’. Within the feeding behavioural state, we noted the kind of resource being fed on: ‘seagrass leaves’ [*P. oceanica* cover: high (80–100%) medium (80–30%) or low (< 30%)] and ‘algae’. It is worth emphasizing that shoals were sampled only within seagrass habitats and surroundings, which precludes any testing of feeding preferences. In addition, we defined three feeding strategies: ‘all-at-once’, when all or more than 75% individuals within the group grazed simultaneously; ‘staggered ’, when 25% or more of the shoal grazed while the rest hovered above the patch (Clip S1); and ‘rotational feeding’, when individuals in the shoal descended to graze while others ascended in an orderly, seemingly synchronised manner (Clip S2). We analysed the proportion of time spent by each shoal in each behavioural state/strategy, which allowed us to compare behaviours between shoals.

### Spatial extent of herbivory

To determine the functional effect of each feeding strategy we used the time spent foraging by an unmoving shoal within an area as a measure of the intensity of grazing. This was estimated by observing how long a shoal remained foraging in one specific point without moving (spot or stationary grazing), while employing a given strategy (e.g. all-at-once, staggered or rotational feeding). Although we could not measure area through video recordings (assess profundity would carry high error), we estimated in the field that area covered by shoals performing stationary grazing usually did not exceed 25 m^2^. Then, we calculated the ‘percent time feeding in a spot’ as the proportion of time shoals performed this focused feeding within each feeding strategy.

### Statistical analysis

#### Individual feeding activity

To determine consumption rates for each length class, we integrated the number of bites (estimated per individual) multiplied by the average duration of a bout and the number of bouts (estimated for every length class separately). Differences in total bites·min^−1^ were evaluated across individual length classes. Class 1 (< 14.6 cm) and class 2 (14.6–20.2 cm) were pooled due to low replication of class 1 (see Figure S1). Bite rates were non-normally distributed so we used non-parametric techniques to test for differences (Kruskal–Wallis and Wilcoxon tests).

#### Shoal composition

To assess if shoals were length-assorted, we tested if ‘shoal length class’ was influenced by ‘individual body length’ using a one-way Anova. This test allowed us to assess if groups belonging to each class were composed of individuals of similar body length. Data was log transformed. Assumptions of normality and homogeneity of variances were inspected visually and fulfilled.

Additionally, we further assessed the homogeneity/heterogeneity of individual lengths within each shoal by calculating the coefficient of variation (CV) within each length class. Also, to determine if length assortment was influenced by ontogeny, we performed a non-parametric test (Kruskal–Wallis) comparing CV across body length classes.

Finally, we evaluated which predictor variables best explained the response variable ‘shoal size’, i.e. number of individual fishes per shoal. We used a Generalised Linear Mixed effects Model (GLMM) with a negative binomial distribution (due to data being overdispersed) and included the variables ‘average shoal body length’, ‘seagrass cover’ (categorical, 3 levels: continuous, fragmented or very fragmented), and ‘location’ as a random factor, to account for the potential shared variance among observations coming from the same location. The predictor ‘seagrass cover’ and the random effect ‘location’ were dropped during variable selection as they did not improve the model (according to the Akaike Information Criterion,^53^). Finally, we used the R package Visreg^54^ to plot the fitted values and regression prediction line of our best-selected model.

#### Shoal foraging behaviour

To check if time using each feeding strategies was different for each shoal class, we performed a Chi-square test. Also, we tested if ‘rotational feeding strategy’ was influenced by ‘shoal size class’ using a Kruskal–Wallis test, given the non-normal distribution of the data. We used post-hoc Wilcoxon pairwise comparisons when significant differences were detected.

#### Spatial extent of herbivory

We used non-parametric Kruskal Wallis tests to assess if the ‘time feeding in a spot’ was influenced by one of the three types of feeding strategies (all-at-once, staggered or rotational) used by shoals. Whenever differences were detected, Wilcoxon pairwise comparisons were also calculated.

## Results

### Individual activity

**Figure 3:**
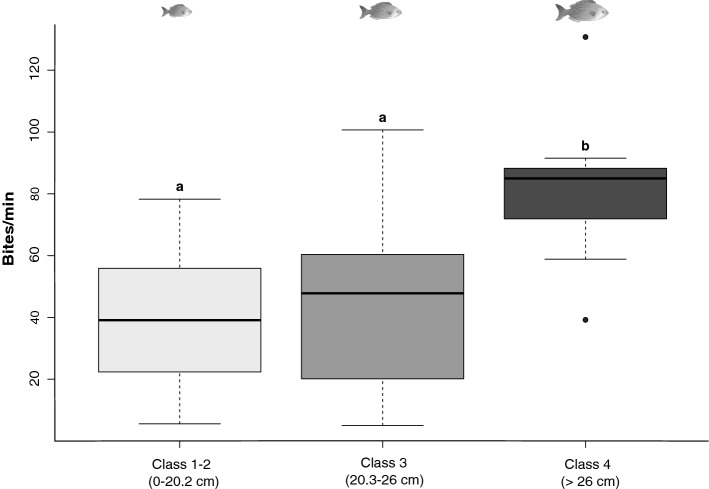
Differences in individual feeding activity (bite rate) of *Sarpa salpa* between length classes*.* Note that length classes refer to the average body length of the fish in a shoal. Each lower case letter indicates significant differences. *Sarpa salpa* image drawn by Tracey Saxby, Integration and Application Network, University of Maryland Center for Environmental Science (https://ian.umces.edu/imagelibrary/).

Rates of seagrass consumption differed significantly between length classes (*p* = 0.005), specifically between the largest (Class 4) and all the smaller classes (Fig. 3). The largest individuals (> 26 cm) consumed, on average, at the rate of > 80 bites/min, more than twice the consumption of smaller length classes (around 40–50 bites/min). These differences were highly significant (See Tables 1 and 2 for non-parametric tests).

**Table 1:** Summary statistics for shoal characteristics and feeding behaviour.

Response variable	Predictor variable	Model	d.f	Statistic	*p* Value
**Shoal characteristics**					
*Length assortment*					
Individual body length	Length class	Linear model	3	F = 532.48	< 0.001***
Coefficient of variation	Length class	Kruskal–Wallis	3	*X*^2^ = 0.657	0.88
*Relationship between body length and shoal size*					
Shoal size	Average body length	General linear model	1	F = 26.74	< 0.001***
**Feeding behaviour**					
Bites (min)	Length class	Kruskal–Wallis	2	*X*^2^ = 10.542	0.005**
Time using feeding strategies	Shoal class	Chi-square	6	*X*^2^ = 217.64	< 0.001***
Time in rotational strategy	Shoal class	Kruskal–Wallis	3	*X*^2^ = 20.693	< 0.001***
Time feeding in a spot	Feeding strategy	Kruskal–Wallis	2	*X*^2^ = 19.816	< 0.001***

*p* values correspond to those provided by each test. D.f = degrees of freedom. Significance values: 0 ‘***’ 0.001 ‘**’ 0.01 ‘*’ 0.05 ‘.’

**Table 2:** Wilcoxon tests used to evaluate pairwise differences between levels of significant effects (see Table 1).

Response variable	Descriptive variable	W	*p* value
**Individual feeding activity by fish length**			
Bites (min)	Class 2–class 3	162	0.608
	Class 2–class 4	10	0.001**
	Class 3–class 4	21	0.007**
**Time spent in rotational strategy by shoals of different sizes**			
Time in rotational strategy	Medium–large	139	0.049*
	Large–very large	78	0.182
	Medium–very large	66	0.004**
**Proportion of time spent feeding in a spot in different feeding strategies**			
Time feeding in a spot	All-at-once–staggered	195	0.002**
	Staggered–rotational	245	0.042*
	All-at-once–rotational	45	< 0.001***

D.f = degrees of freedom. Significance values: 0 ‘***’ 0.001 ‘**’ 0.01 ‘*’ 0.05 ‘.’

### Shoal composition

**Figure 4:**
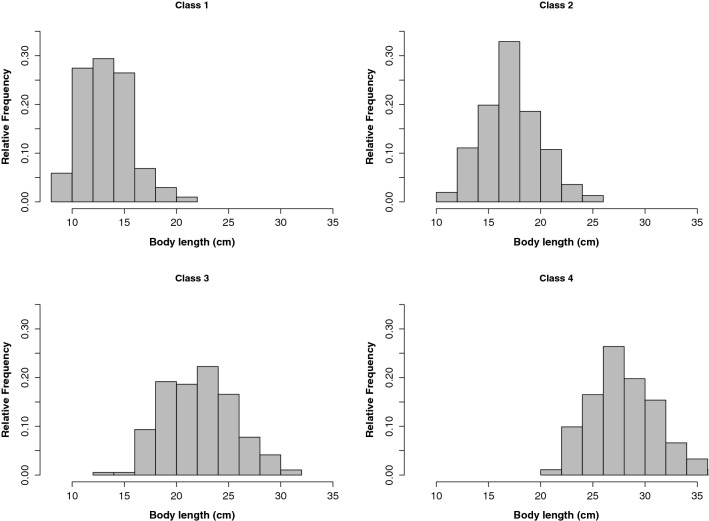
Frequency distribution of *Sarpa salpa* individuals within shoals of different body length classes. Shoals are strongly length assorted, with very low dispersion around the average body length of the shoal. Body length classes: Class 1 (< 14.6 cm), Class 2 (14.6–20.2 cm), Class 3 (20.3–26 cm) and Class 4 (> 26 cm).

All shoals were strongly size-assorted as can be seen in the narrow frequency range of body-length histograms (Fig. 4). For instance, shoals belonging to ‘Class 1’ (i.e. shoals with an average body length < 14.6 cm) were principally composed of individuals smaller of 15 cm, and those belonging to ‘Class 2’ were composed of individuals from 15 to 20 cm, and so on. These trends were supported by linear models that showed that ‘shoal length class’ was a significant predictor of ‘individual body length’ (Table 1, see Supplementary Fig. S3). The average coefficient of variation for all recorded shoals was 13%, indicating that length variation between individuals within a shoal was low. In addition, coefficients of variation did not differ between shoal length classes.

**Figure 5:**
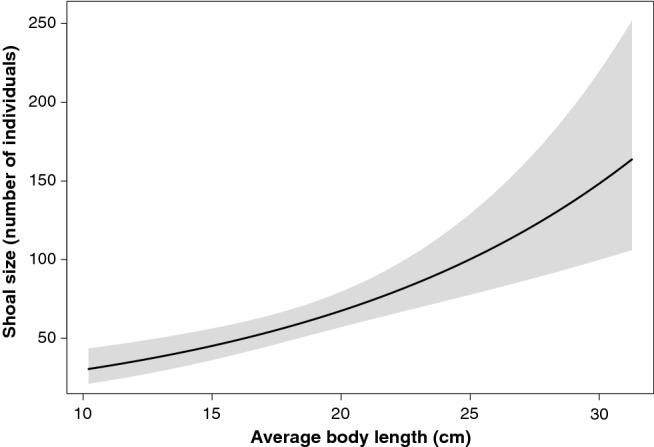
Shoal size of *Sarpa salpa* (number of individuals) increased with average body length (cm). The solid line indicates the best fit of the GLM and the grey band corresponds the 95% confidence interval.

Shoal size (number of individuals) increased significantly with shoals’ average body length (Table 1). The length of fish in shoals of < 50 individuals was typically < 15 cm. In contrast, the largest shoals (> 150 individuals) had fish that were typically > 26 cm (Fig. 5).

### Shoal foraging behaviour

**Figure 6:**
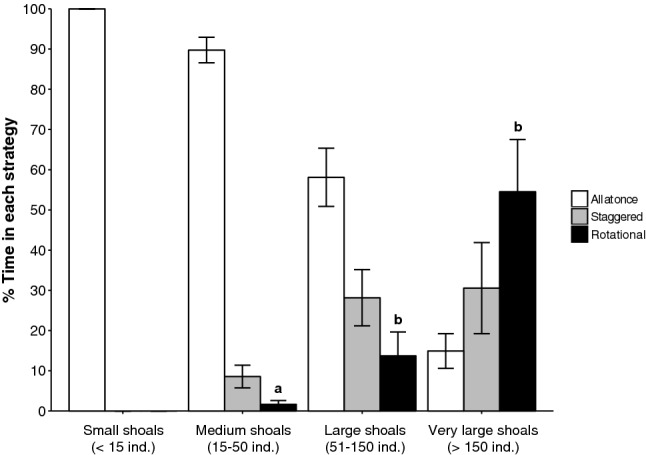
Proportion of time spent on different feeding strategies assessed for each shoal size class of *S. salpa*. Shoal sizes: small (< 15 individuals), medium (15–50 individuals), large (50–150 individuals) and very large (> 150 individuals). Each lower case letter indicates significant differences in rotational grazing.

Feeding strategies varied significantly with shoal size (*p* value < 0.001, Fig. 6). The smallest shoals (< 15 individuals) fed exclusively with an ‘all-at-once’ strategy, while the frequency of ‘staggered’ and ‘rotational’ strategies increased as shoal size increased. At the extreme, the largest shoals (> 150 individuals) engaged in ‘all-at-once’ feeding only 15% of the time, while the ‘rotational’ strategy was used > 50% of the time. Non-parametric tests confirmed that the rotational strategy was used significantly more by larger shoals (Table 2).

### Spatial extent of herbivory

**Figure 7:**
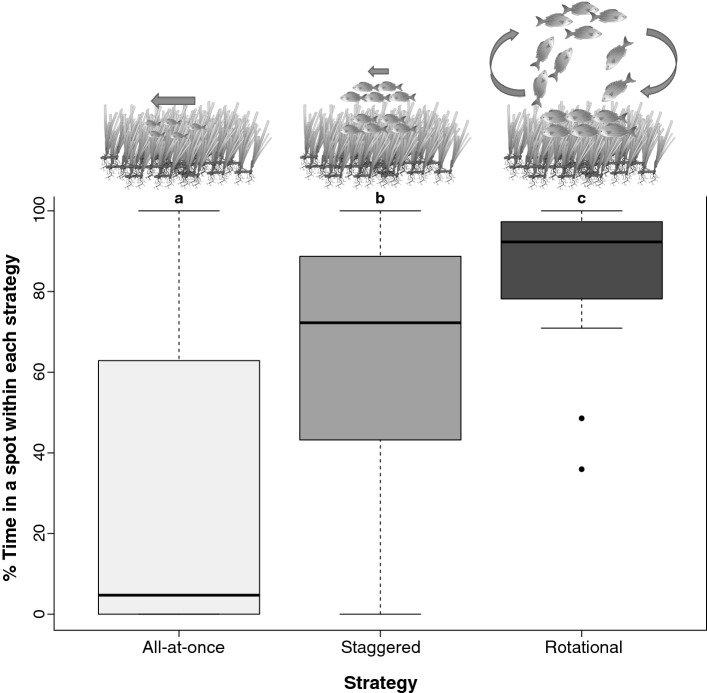
Proportion of time spent in feeding in a spot for each feeding strategy. Shoals using staggered and rotational strategies were much more stationary in their feeding than shoals using the all-at-once strategy. Each lower case letter indicates significant differences. *Sarpa salpa* image drawn by Tracey Saxby, Integration and Application Network, University of Maryland Center for Environmental Science (https://ian.umces.edu/imagelibrary/).

When shoals fed with an ‘all-at-once’ strategy, they wandered continuously through the meadow spending less than 10% of their time feeding at any chosen spot. In contrast, when shoals used the ‘staggered’ strategy spent up to 75% of their time feeding in a single spot. This was even more pronounced for the ‘rotational’ strategy, when shoals spent up to 90% of their time feeding within the same spot (Fig. 7, Table 2).

## Discussion

The distribution and persistence of *Sarpa salpa* herbivory on *Posidonia oceanica* meadows is linked to the size of the fish in more ways than one. At its simplest, herbivory is a mere function of size—the larger the individual, the more it consumes. Also, as individuals age and their metabolisms change, they may completely switch diet^55–57^. Group foraging strategies add yet another factor to consider while evaluating the effects herbivorous fish have on seagrass meadows. If groups are length-assorted and group size grows as individuals grow, the overall impact of groups should increase disproportionately with age, as a function of both an increase in size and of number. Finally, as groups grow, they show increasingly complex behaviours that allow them to feed more effectively together. Large groups employ potentially cooperative strategies where individuals within a shoal feed sequentially or rotationally, potentially giving individuals access to more nutritional plant tissues by intensively grazing a relatively small area. The full extent of herbivory is therefore a function of the number of fishes and resource availability^58^ but is also a function of size-mediated individual and collective feeding behaviour (Fig. 1). Individual feeding behaviour and group-level feeding behaviour (a result of body length-assortment, shoal size and feeding strategies), act together to determine the distribution and intensity of herbivory across the landscape.

Shoal formation in *S. salpa* was strongly size assorted – individuals of the same body length tended to stick together. This is not uncommon across taxa from insects^59^, amphibians^60^, birds^61^, mammals^62^ and many fish^8^ whose groups separate based on size, standing in for a range of important life-history traits and all the metabolic, behavioural and social changes that age brings. As they grow, *S. salpa*, like many other fish, show major changes in their diets, shifting from algae to seagrass^37,44^. Even though our observations focused on seagrass meadows and their surroundings alone, we found a tendency for the smallest individuals to spend relatively longer foraging on macroalgae within the landscape, while the largest individuals fed exclusively on seagrass (see Supplementary Fig. S4). Ontogenetic habitat specialisation has been observed in other groups that use distinct resources as their nutritional requirements changed through life^63^. Even though we have not tested it in this study, the effects of predation, past or present, could also influence body length assortment. The oddity effect, where anomalous sizes face a higher risk of predation and are less competitive than median sizes, is a strong evolutionary driver for group composition converging to individuals of similar sizes^8,12,13^. Additionally, as individuals grow, they may also grow in their ability to compete with conspecifics within the group. As we discuss below, larger groups may also show cooperative behaviours that require coordination and specialisation. Younger, less experienced individuals may not be able to participate in these behaviours, and may find themselves excluded from or disadvantaged in groups of larger individuals. For instance, marine grazers develop a range of distinct strategies relative to diving depth, that depend on their physical capacities and their target prey^63–66^ that end up determining the assortment of groups by size classes. For instance, a strong relationship between body size and diving behaviour has been found in California sea lions^63^, which determines where and how deep individuals can dive, effectively segregating individuals by their size.

In this study, we show that the number of *S. salpa* individuals per shoal increased with individual fish size, with large individuals commonly forming shoals higher than a hundred of individuals. The number of individuals per shoal could not be explained by resource availability (seagrass cover), given that the main resource of *S. salpa* (i.e. *P. oceanica*) is highly abundant in the study areas. Theory suggests that grouping behaviour reduces predation risk for individuals by diluting the probability of predation for every individual and by increasing overall group vigilance^7–9^. However, in our study system few extant predators serve a realistic threat to adult *S. salpa*, particularly to the largest individuals, which dominated the large shoals. We cannot discount the possibility of fish responding to past predation^67^; previous studies show that other herbivores, such as sea urchins, still experience fear from predation even after having reached their size refuge, when predation is no longer a significant risk^27^. Nevertheless, the results from the present study suggest that there may be clear advantages to foraging in large shoals—possibly linked to acquiring high quality food that can only be accessed by facilitative foraging (see below). Smaller individuals most likely cannot access these shoals because of intraspecific competition or because they are unable to participate in the complex foraging behaviours that large shoals show.

It is difficult with our observational study to distinguish between the relative importance of ontogenetic diet shifts, predation risk or competitive abilities as drivers of size assortment. However, our description of grouping characteristics of *S. salpa* show that body length is the main factor in shoaling composition, being closely linked to shoal size and ultimately influencing shoal feeding behaviour. In addition, the strong separation we recorded between lengths has important consequences for the way herbivory is distributed across the landscape. This is magnified by the fact that foraging behaviour changed with increasing shoal size. It is true that, because of the strong length assortment of shoals and the relationship between body length and shoal size, it is difficult to separate how much these variations in foraging behaviour are a result of ontogeny and how much they are related to the size of the group itself. The fact that the smallest shoals (also composed of the smallest individuals) fed considerably on algae is probably a result of ontogenetic dietary requirements. However, the shoal feeding strategies—all-at-once, staggered or rotational feeding—appear to be more a function of shoal size, with the latter two employed much more frequently as shoal size increased. The largest shoals spent more than 50% of their time in rotational grazing. Rotational grazing is a unique cooperative foraging where the entire shoal appeared to cycle in the same place with individuals feeding sequentially within the meadow. This strategy requires a certain degree of behavioural coordination and probably serves to ensure that all individuals within a shoal get access to preferred patches of a meadow or basal portions of *Posidonia oceanica* leaves. Given the length of *P. oceanica* leaves (often up to a metre), other feeding strategies (e.g. all-at-once) are unlikely to gain access to these nutrient-rich basal leaves^68^. A focused spot-foraging strategy may allow individuals to continuously crop the canopy down to its nutritious base. It is possible that rotational grazing can emerge only beyond a certain shoal size and may be inefficient below this size. It may also require a certain degree of specialisation within the group, where individuals take on initiator, facilitator and vigilance roles as the group rotates within the patch. It is not unusual for such specialisation to emerge as group sizes increase in species as far apart as insects and humans^32,69,70^.

At the individual level**,** *S. salpa* also altered its feeding activity by modifying its consumption rate across length classes. Larger fish consumed more per minute than smaller individuals (Fig. 3), and are likely to be more efficient grazers^8,28,71^. This may be linked to rotational feeding, that was especially prevalent among large individuals in large shoals, and may be a result of the restricted grazing time this strategy implies. Large individuals, in large shoals, may need to increase their consumption rate to maximise the amount of intake in each feeding bout. The positive relationship between mouth gape size and body length^47^ additionally improves the efficiency of food intake per bout, considerably increasing leaf offtake compared to smaller shoals.

Smaller shoals distributed their herbivory more-or-less uniformly across the meadow, using an ‘all-at-once’ feeding strategy, where all fish grazed together, moving quickly and widely through the landscape and covering large areas without focusing their herbivory on any one location. In contrast, larger shoals were much more localised in their movement, spending longer periods foraging on a single patch of seagrass. This ‘spot foraging’ was most pronounced for rotational foraging strategies, when the shoal spent an average of 90% feeding at the same location. These large shoals fed on a remarkably small area of the meadow (usually not exceeding 25 m^2^, pers. obs.) in relation to the size of the shoal. Such a sedentary feeding strategy may be energetically and nutritionally more efficient for large shoals, reducing the time spent travelling between feeding patches, and extracting as much as possible from a patch once it had been located. However, this concentrates herbivory and is capable of significantly affecting the vegetation, reducing seagrass shoot length within the patch within a few minutes (pers. obs.). In the presence of large *S. salpa* foraging aggregations, the habitat could therefore be subject to patchy, but very intense herbivory pressure^50^ and this could be particularly important for those meadows that host higher abundances or large shoals of fish^47^. While the overall abundance of *S. salpa* and their resources are certainly among the main drivers of overall herbivory^58^, our study highlights how individual and collective behaviours can be critical in mediating this impact. Although, at current population densities, *S. salpa* are unlikely to be responsible for large scale seagrass collapses, how shoals forage may create mosaics of grazing—maintaining a heterogeneous landscape that could have further consequences for the way other species use it. Indeed, it has been documented that this way of grazing can be important for plant performance^39,50^, meadow diversity and for the distribution of other functions like predation^40^.

Foraging aggregations can have major consequences for seagrass meadows and other plant-based ecosystems. Our results show that, for group foraging herbivores, size matters in more ways than one. Apart from simple allometric, ontogenetic and morphological changes in feeding as individuals grow, the impact of herbivores scale with their number as well as how they behave—individually as well as in groups. As groups grow both in length and number, increasingly specialised and increasingly efficient foraging strategies may emerge that can further increase the impacts these groups have on the ecosystems they graze in. A complete understanding of how grazing is distributed across the landscape requires an appreciation of size, number and behaviour of herbivores, and how each of these change with ontogeny.

## Ethical approval

All applicable international, national, and/or institutional guidelines for behaviour research on animals were followed. All procedures performed in studies involving animals were in accordance with the ethical standards of the institution at which the corresponding author is based.

## Supplementary information

Supplementary file1

Supplementary file2

Supplementary file3

## Data Availability

The datasets collected, graphed, and analysed are available from the corresponding author upon request.
