# Supplementary material for: The dominant seagrass herbivore Sarpa salpa shifts its shoaling and feeding strategies as they grow
Source: Sci Rep. 2020 Jun 30;10:10622. doi: 10.1038/s41598-020-67498-1 (PMC7327017; doi:10.1038/s41598-020-67498-1)
Supplement: Supplementary file 1 — Supplementary file1 [file 41598_2020_67498_MOESM1_ESM.pdf]

The dominant seagrass herbivore *Sarpa salpa* shifts its shoaling and feeding strategies as they grow

Xavier Buñuel*, Teresa Alcoverro, Jordi F. Pagès, Javier Romero, Juan M. Ruiz, Rohan Arthur

Correspondence to Xavier Buñuel

https://orcid.org/0000-0001-9064-3912

Adress: Accés a la cala Sant Francesc 14, 17300 Blanes, Spain

Phone: +34638476262

e-mail: xbunuel@ceab.csic.es

Supplementary material

Figure Legends

Fig.S1. Abundance of a) shoal length classes for each shoal size and b) shoal sizes for each location.

Fig S2. Frequency distribution of *Sarpa salpa* a) individual body lengths and b) shoal sizes.

Fig. S3. Body length (cm) of individuals composing each shoals' body length classes. Body length classes: Class 1 (<14.6 cm), Class 2 (14.6-20.2 cm), Class 3 (20.3-26 cm) and Class 4 (>26 cm).

Fig. S4. Proportion of time spent feeding on different resources for each body length class of *Sarpa salpa*. Only shoals recorded in areas with both seagrass and algae are included. Body length classes: Class 1 (<14.6 cm), Class 2 (14.6-20.2 cm), Class 3 (20.3-26 cm) and Class 4 (>26 cm). Note that shoals containing individuals of the largest length class (>26 cm) fed exclusively on seagrass while the smallest length class (<14.6 cm) spent 35% of their time feeding on algae.

Clip S1. Staggered feeding strategy clip.

Clip S2. Rotational feeding strategy clip.

Fig S1

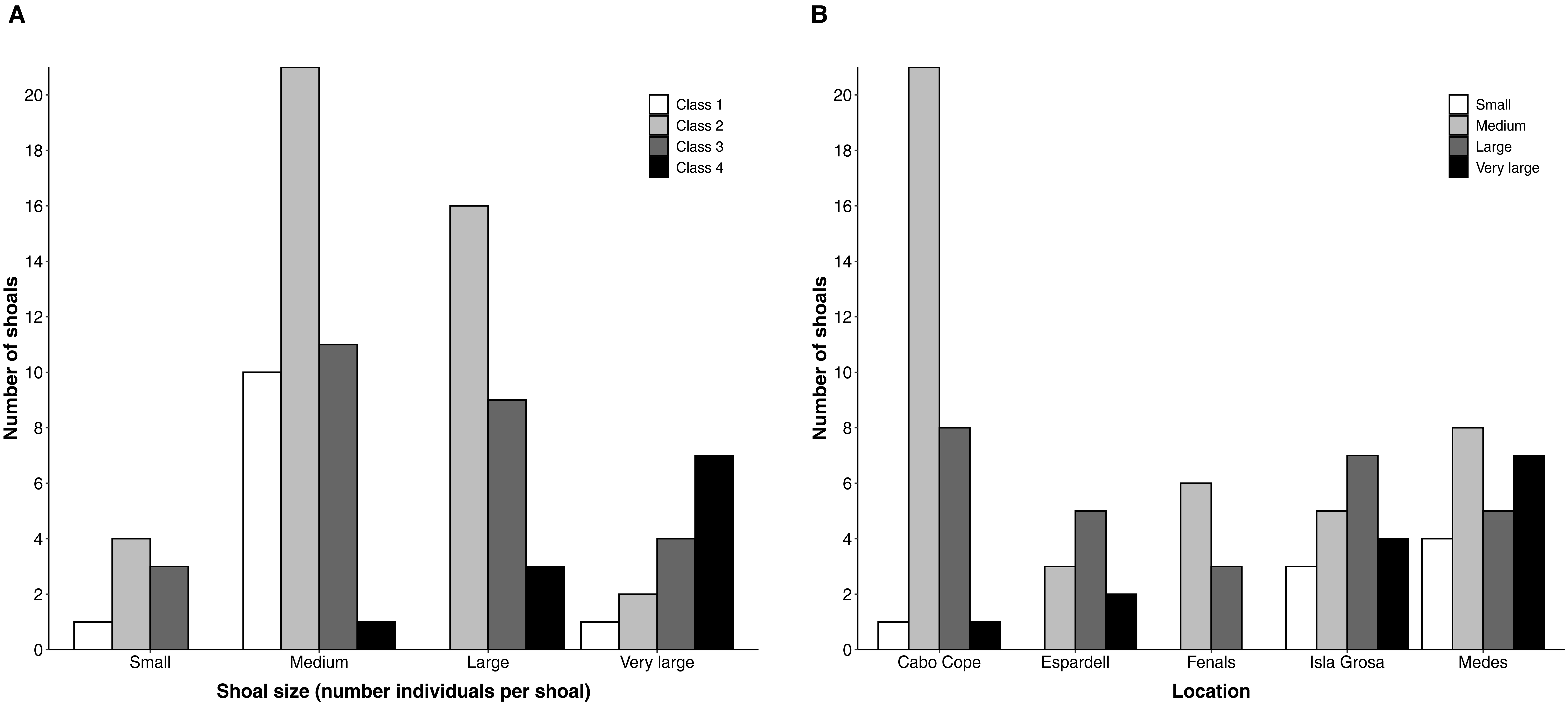

A
B
Number of shoals
0
2
4
6
8
10
12
14
16
18
20
Class 1
Class 2
Class 3
Class 4
Small
Medium
Large
Very large
Shoal size (number individuals per shoal)
Cabo Cope
Espardell
Fenals
Isla Grosa
Medes
Location

Fig S2

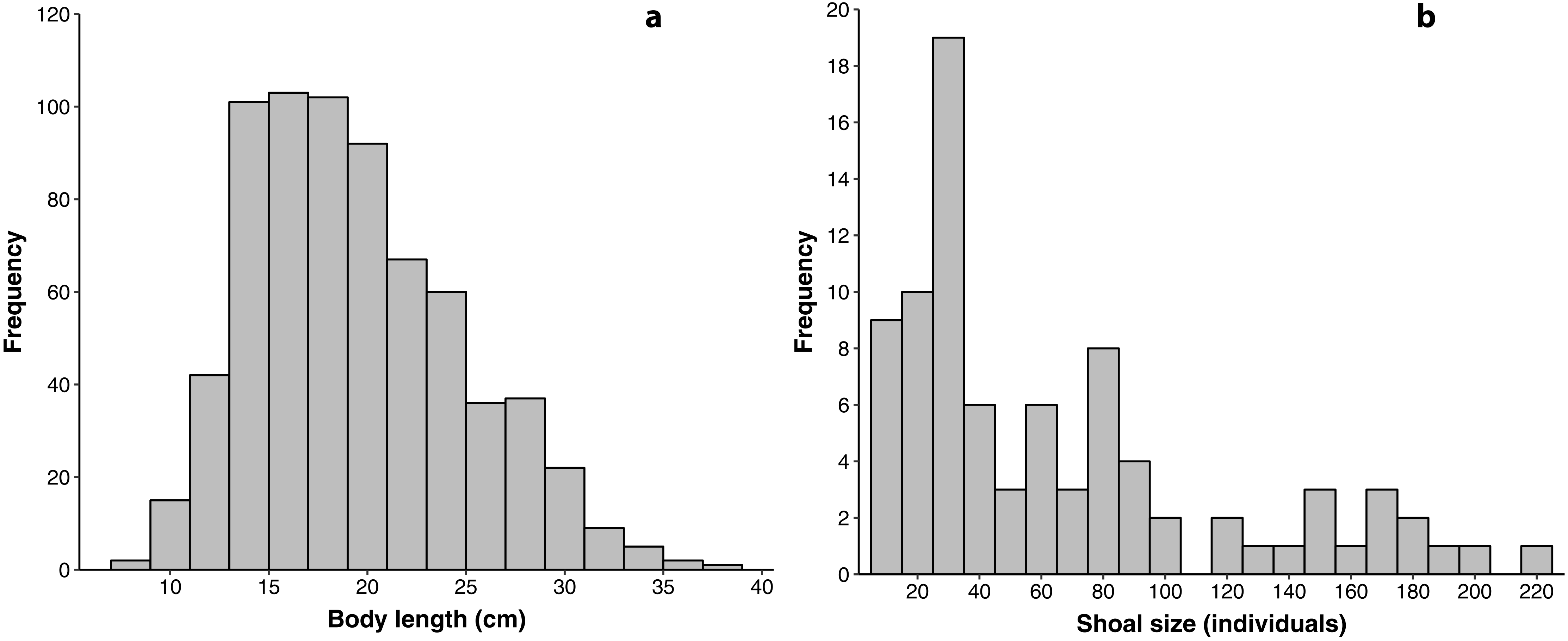
a
b
Frequency
Body length (cm)
Shoal size (individuals)
0
20
40
60
80
100
120
10
15
20
25
30
35
40
0
2
4
6
8
10
12
14
16
18
20
20
40
60
80
100
120
140
160
180
200
220

Fig S3

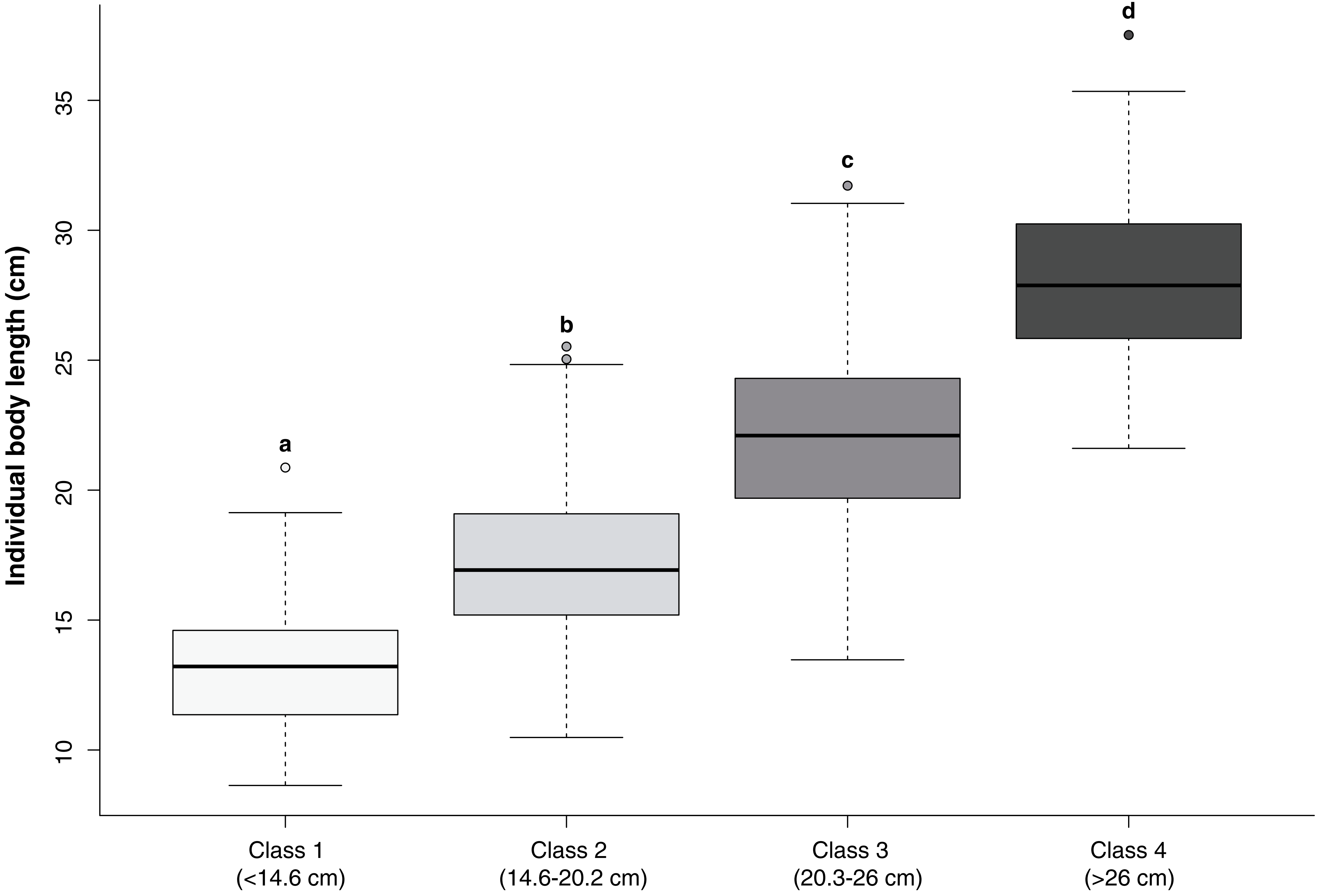

Individual body length (cm)
10
15
20
25
30
35
a
b
c
d
Class 1
(<14.6 cm)
Class 2
(14.6-20.2 cm)
Class 3
(20.3-26 cm)
Class 4
(>26 cm)

Fig S4

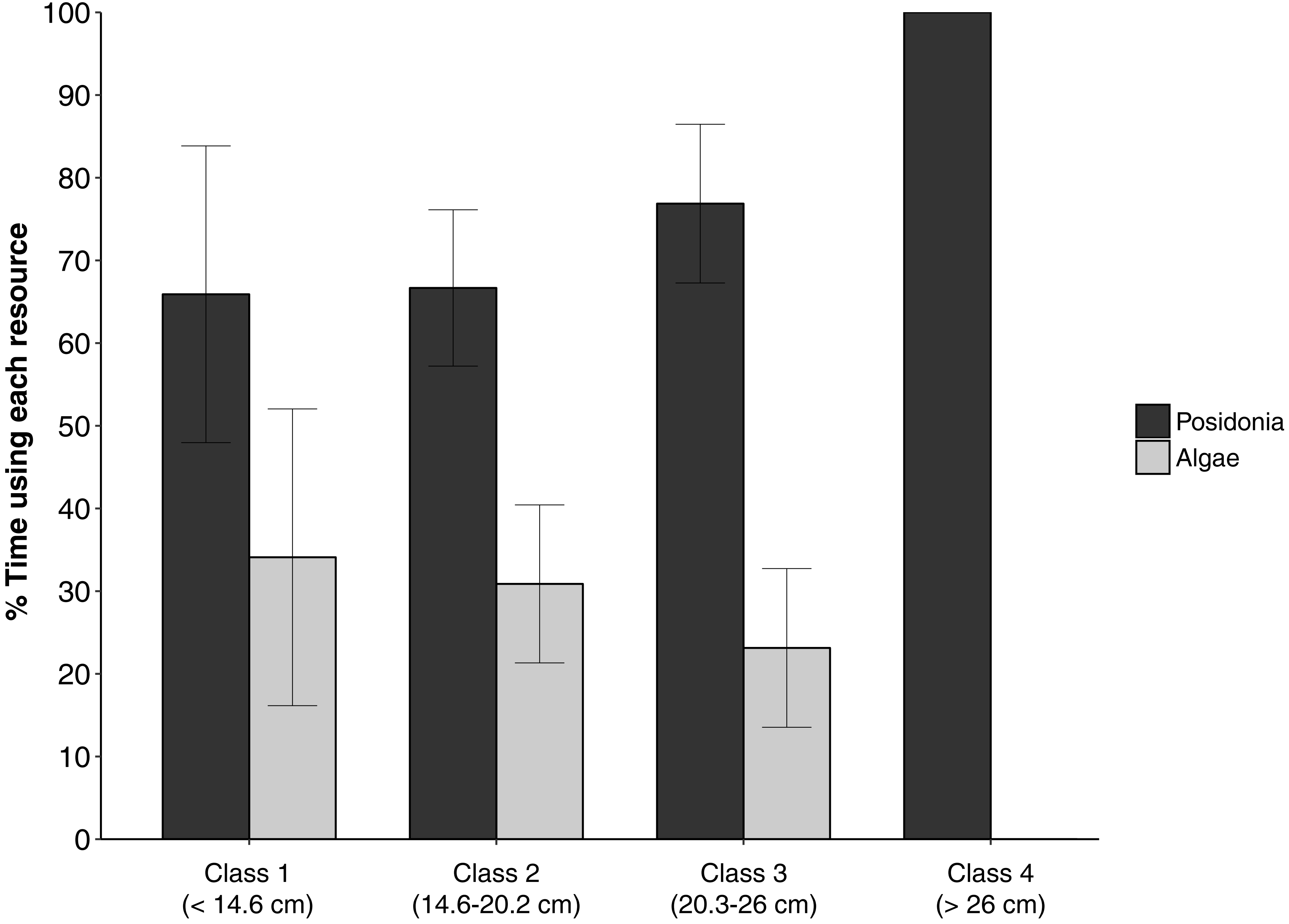

% Time using each resource
100
90
80
70
60
50
40
30
20
10
0
Class 1
(< 14.6 cm)
Class 2
(14.6-20.2 cm)
Class 3
(20.3-26 cm)
Class 4
(> 26 cm)
Posidonia
Algae
